# Ocular Manifestations in Dogs With Facial Nerve Dysfunction

**DOI:** 10.1111/vop.70219

**Published:** 2026-07-01

**Authors:** L. Gaztelu, M. Leiva, T. Peña, J. Ríos

**Affiliations:** ^1^ Servei d'Oftalmologia, Fundació Hospital Clínic Veterinari Universitat Autònoma de Barcelona Bellaterra Spain; ^2^ Departament de Medicina i Cirurgia Animals, Facultat de Veterinària Universitat Autònoma de Barcelona Bellaterra Spain; ^3^ Biostatistics Unit, School of Medicine Universitat Autònoma de Barcelona Barcelona Spain

**Keywords:** canine, corneal ulcer, facial paralysis, neurogenic keratoconjunctivitis sicca, neuroparalytic keratitis, ulcerative keratitis

## Abstract

**Objective:**

To characterize the spectrum and frequency of ocular abnormalities associated with facial nerve dysfunction (FND) in dogs and to assess clinical outcomes, with specific comparison between brachycephalic (BC) and non‐brachycephalic (NBC) breeds:

**Methods:**

Medical records of dogs with FND evaluated between 2009 and 2023 at the Ophthalmology Service of the Hospital Clínic Veterinari, Universitat Autònoma de Barcelona, were retrospectively reviewed.

**Results:**

Seventy‐four dogs (84 eyes) were included, with French Bulldog being the most frequently affected breed (20; 27.0%). Facial nerve paralysis affected 63.1% of eyes, whereas paresis affected 36.9%. The main etiologies were idiopathic (14.9%), iatrogenic (13.5%), and otitis media/interna (12.2%). Corneal sensitivity and Schirmer tear test I were significantly reduced in affected eyes, without significant differences between skull types. Ulcerative keratitis occurred in 61.9% of eyes, being more frequent and severe in BC dogs (*p* = 0.007). Facial function recovered in 47.6% of dogs. Recovery was unaffected by age or skull type, differed significantly by sex (*p* = 0.043), and tended to be higher in dogs with paresis (*p* = 0.075). Worse ocular outcomes were linked to lower esthesiometry values (*p* = 0.023) and to the presence of anterior uveitis (*p* = 0.037). At the end of the study, 14 eyes (17.6%) were enucleated.

**Conclusions:**

FND carries a guarded prognosis in dogs, with fewer than half achieving functional recovery. Ocular surface disorders, particularly neurogenic keratoconjunctivitis sicca and neuroparalytic ulcerative keratitis, were frequent and often led to poor outcomes, especially in BC dogs. Early ophthalmic management is crucial to preserve vision.

## 1 Introduction

The facial nerve is a mixed cranial nerve that provides three types of fibers: motor fibers to the muscles of facial expression and to the stylohyoideus, the caudal belly of the digastricus, the stapedius and the platysma muscles; parasympathetic fibers to the lacrimal gland, nasal mucosal glands and certain salivary glands; and sensory fibers for taste [1]. The anatomy of the facial nerve has been extensively described [2, 3, 4, 5]. Briefly, the motor and parasympathetic fibers originate from their respective nuclei in the medulla oblongata. They exit the brainstem as two distinct roots, a larger motor root and a smaller sensory, parasympathetic root, known as the *nervus intermedius*, which joins the vestibulocochlear nerve to enter the internal acoustic meatus. They then course through the facial canal of the petrous part of the temporal bone, forming a sharp bend ‐the genu‐ at the level of the geniculate ganglion. It is in this ganglion that the cell bodies of the sensory fibers for taste are located, which temporarily join the course of the motor and parasympathetic fibers. From this point, the fibers follow different courses. Parasympathetic and gustatory fibers branch off intracranially. Parasympathetic fibers for the mandibular and sublingual salivary glands, together with taste fibers, leave through the chorda tympani before the facial nerve exits the skull. Conversely, parasympathetic fibers for the lacrimal and nasal glands leave the facial canal as the greater petrosal nerve, which synapses in the pterygopalatine ganglion before reaching their target glands. Motor fibers continue through the facial canal, cross the middle ear cavity ‐where the nerve is exposed‐, and exit the skull via the stylomastoid foramen to innervate the muscles of facial expression.

Facial nerve dysfunction (FND) in dogs has been associated with a variety of pathologic and traumatic conditions. Idiopathic facial nerve paralysis is the most common cause, with a reported prevalence ranging from 29.5% [3] to 74.7% [6]. It has also been associated with primary disease processes (middle ear disease including otitis and polyps [2, 7], retrobulbar cellulitis or abscessation [8], hypothyroidism [9, 10] with secondary hyperlipidemia [11], diabetes mellitus [12], myasthenia gravis [13], polyradiculoneuritis [13], insulinoma [14], cranial or central nervous system (CNS) cysts and neoplasms [15, 16, 17, 18, 19], and acute myeloid leukemia [6, 20]), iatrogenic trauma (total ear canal ablation with lateral bulla osteotomy [TECA‐LBO] [21, 22, 23, 24, 25, 26], parotidectomy [27], surgical approach to the middle fossa [28], removal of retrobulbar foreign body [29]), snake envenomation [30], tick paralysis [31], aspergillosis‐induced petrous temporal bone osteomyelitis [32], cryptococcosis [33], suspected rabies [34], and drug toxicity (potentiated sulfonamides) [35, 36].

In neurological terminology, paresis refers to a partial loss of motor function, with reduced but not absent muscle strength and movement, whereas paralysis denotes a complete loss of motor function. These definitions are consistent across both human and veterinary medicine and are widely used in neuro‐ophthalmological contexts.

Clinical signs of FND include facial asymmetry, loss of lip tone, ear drooping in some dogs (not in cats or dogs with taut ear cartilage), and absence of spontaneous blinking. Denervation of the salivary glands may also result in more viscous saliva [13]. Ophthalmic consequences are particularly important: loss or reduction of blink reflex, impaired eyelid closure, and decreased tear production predispose the cornea to desiccation, ulceration, infection, and, in the worst scenario, perforation. Such complications may lead to permanent vision loss and significantly compromise animal welfare if not promptly recognized and treated.

Although isolated descriptions exist, to the best of the authors' knowledge, no studies have specifically reported the incidence of ocular manifestations in dogs with FND. Given the potential of these changes to cause pain, blindness, and irreversible ocular damage, their characterization is clinically relevant. Therefore, the aims of this study are to describe the prevalence of the ocular findings associated with FND in dogs and to report outcomes, with particular attention to differences according to cranial conformation. Based on the brachycephalic ocular syndrome, authors hypothesize that brachycephalic (BC) dogs experience more severe ocular disease and a worse prognosis than non‐brachycephalic (NBC) dogs, requiring more intensive monitoring and treatment.

## 2 Materials and Methods

### 2.1 Ethical Approval

This study complies with the Guidelines for Ethical Research in Veterinary Ophthalmology (GERVO) and, due to its retrospective and purely clinical nature, without any intervention beyond routine diagnostic and therapeutic procedures, formal approval from an ethics committee was not required.

### 2.2 Inclusion Criteria and Data Collection

Medical records of dogs evaluated by the Ophthalmology Service of the Hospital Clínic Veterinari, Universitat Autònoma de Barcelona, between 2009 and 2023, were retrospectively reviewed. Inclusion criteria were a confirmed diagnosis of FND and a complete ophthalmic and neurologic examination performed by a board‐certified veterinary ophthalmologist and neurologist or a resident in training, respectively. All dogs underwent a comprehensive ophthalmic examination, which included distant evaluation, Schirmer tear test I (STT‐I; MSD Animal Health), neuro‐ophthalmic assessment, slit‐lamp biomicroscopy (SL‐17; Kowa Company Ltd.), rebound tonometry (Tonovet Plus; Icare Findland Oy), indirect ophthalmoscopy (Omega 500; Heine), and fluorescein staining (FluoroTouch; Madhu Instruments). Additional tests, such as esthesiometry (Cochet‐Bonnet esthesiometer; Luneau Ophtalmologie) or corneal cytology, were performed when clinically indicated. The neurologic examination included the evaluation of the mental status, gait and posture evaluation, postural reactions, cranial nerves examination, spinal reflexes, and sensory evaluation.

Collected data included signalment, laterality, and presumed cause of FND; diagnostic tests performed to determine the etiology (blood work including thyroid hormones analysis, otoscopy, and advanced imaging); presence of other neurologic abnormalities; concurrent ocular or systemic diseases; medical and surgical treatments; follow‐up period; and clinical outcome regarding recovery of facial function and ocular signs. Special attention was given to BC status, STT‐I results, corneal sensitivity (when available), the depth and characteristics of any ulcerative keratitis, and signs of anterior uveitis.

Cases of FND were classified as idiopathic when no cause could be identified despite completion of a full diagnostic protocol, whereas cases in which the diagnostic workup was incomplete were categorized as having an unknown etiology.

### 2.3 Grading System for the Ophthalmic Findings

A STT‐I value below 15 mm/min was considered reduced. Bilaterally decreased results in patients with unilateral facial dysfunction, or in those with a previous diagnosis of keratoconjunctivitis sicca (KCS) or aqueous‐deficient dry eye (ADDE), were interpreted as unrelated to the neurological deficit. In contrast, in unilaterally affected patients with ipsilateral low STT‐I values – particularly when accompanied by xeromycteria‐ were regarded as neurogenic in origin, leading to a diagnosis of neurogenic KCS (nKCS). Esthesiometry was recorded in centimeters to provide consistency with previous veterinary reports [37, 38]. Corneal ulceration was categorized as superficial (including erosions, epithelial and anterior stromal ulcers), deep (including mid‐stromal ulcers to descemetoceles), or perforations.

### 2.4 Outcome Description

Recovery of FND was defined as *complete* when motor activity, particularly the blink reflex, was fully restored, and *partial* when some degree of improvement was observed without complete restoration. Similarly, ocular outcome was considered *satisfactory* when there was complete resolution of clinical signs, *partially satisfactory* when improvement occurred without full resolution, and *unsatisfactory* when no improvement or worsening was observed. For statistical purposes, partially satisfactory and unsatisfactory ocular outcomes were grouped together and classified as unfavorable.

### 2.5 Statistical Analysis

All results were described by skull morphology and recovery. For comparison by skull morphology, categorical variables were described by means absolute frequencies and percentages and analyzed using the Fisher's Exact Test. Continuous variables were described using median and 25th and 75th percentiles, with a Mann–Whitney U test or Wilcoxon paired test for statistical comparisons between unpaired or paired groups. The effect of brachycephaly on follow‐up until complete or partial recovery from facial dysfunction was assessed using the Log‐Rank test, showing recovery time using median estimates by the Kaplan–Meier method. A *p*‐value < 0.05 was considered statistically significant and all statistical analyses were made using IBM SPSS Statistics (Version 27, Armonk, NY) software.

### 2.6 Artificial Intelligence Generated Content

ChatGPT was used to assist in generating the figures illustrating some of the results.

## 3 Results

### 3.1 Signalment

Overall, 74 dogs met the inclusion criteria. Sixty‐four were affected unilaterally and 10 bilaterally, resulting in 84 affected eyes. The median age at presentation was 7.45 years (range 1–14 years). The cohort included 29 intact males (39.2%), 13 neutered males (17.6%), 17 intact females (23.0%), and 15 spayed females (20.3%). French Bulldog was the most frequently affected breed (20; 27.0%), followed by mixed‐breed (10; 13.5%), Golden Retriever (5; 6.6%), Boxer (5; 6.6%), Maltese (4; 5.4%), Cavalier King Charles Spaniel (3; 4.1%), West Highland White Terrier (3; 4.1%), Pug (2; 2.7%), and Rottweiler (2; 2.7%). Sixteen additional breeds were represented by a single individual each. According to skull conformation, 35 dogs were classified as BC (40 affected eyes) and 39 as NBC (44 affected eyes); both groups included 5 bilateral cases.

Table 1 summarizes the main results for BC and NBC dogs, allowing direct comparison between groups.

**TABLE 1:** Comparison based on brachycephaly.

	Brachycephalic	Non‐brachycephalic	*p*
Severity (*n*)			0.26
Paralysis	28	25	
Paresis	12	19	
Etiology (*n*)			**0.009**
Idiopathic	8	3	
Otitis	7	2	
Iatrogenic	7	3	
MUO	2	4	
Trauma	1	3	
Metabolic	0	2	
Other	0	4	
Ocular abnormalities			
nKCS (*n*)	9	15	0.339
Esthesiometry (median, cm)	0.50	1.25	0.391
Ulcerative keratitis (*n*)			
Ulcer depth (*n*)	31	21	**0.007**
Superficial	26	18	**0.015**
Deep	3	3	
Perforation	2	0	
Anterior uveitis (*n*)	14	11	0.348
FND recovery	11	19	0.131
Time (days)	155.95	180	0.542
Ocular outcome			
Satisfactory (*n*)	8	13	0.269
Enucleation (*n*)	9	5	0.243

*Note:* The data are analyzed using Fisher's exact test for qualitative variables and Mann–Whitney U test for quantitative variables. Significant *p*‐values are shown in bold.

Abbreviations: FND, facial nerve dysfunction; MUO, meningoencephalitis of unknown origin; nKCS, neurogenic keratoconjunctivitis sicca.

### 3.2 Facial Nerve Dysfunction

Paralysis was recorded in 53 eyes (53/84; 63.1%), occurring in 28 BC (28/40; 70%) and 25 NBC (25/44; 56.8%) eyes. Paresis was present in 31 eyes (31/84; 36.9%), including 12 BC (12/40; 30.0%) and 19 NBC (19/44; 43.2%) eyes.

Magnetic resonance imaging (MRI) was performed in 33 cases (35 affected eyes), while computed tomography (CT) was used in 2 cases (2 eyes). Advanced imaging contributed to establishing an etiological diagnosis in all imaged cases (37 eyes), including idiopathic disease (11 eyes), otitis media/interna (8 eyes), meningoencephalitis of unknown origin (MUO; 6 dogs, 8 eyes), and iatrogenic causes (6 eyes). Additional isolated diagnoses included nonsurgical trauma, intracranial mass, stroke, and eosinophilic meningitis.

Overall, an underlying etiology was identified in 46 dogs (62.2%), while it remained unknown in 28 cases (37.8%). FND was idiopathic in 11 dogs (14.9%), secondary to iatrogenic causes in 10 (13.5%), otitis media/interna in 9 (12.2%), and MUO in 6 (8.1%). Trauma, metabolic disorders, and other less common causes accounted for 4 (5.4%), 2 (2.7%), and 4 (5.4%) cases, respectively.

**FIGURE 1:**
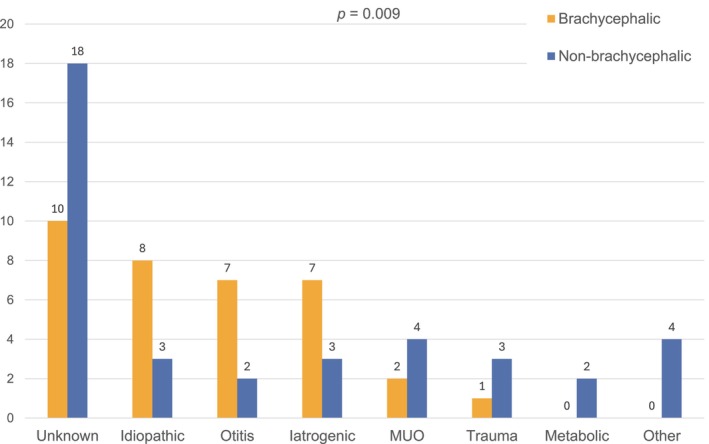
Etiologic distribution of facial nerve dysfunction in brachycephalic (BC) and non‐brachycephalic (NBC) dogs. Idiopathic, otitis‐related, and iatrogenic‐related cases were more common in BC dogs, while MUO‐related, traumatic, and other causes showed a higher representation in NBC dogs. MUO, meningoencephalitis of unknown origin.

The distribution of etiologies according to skull conformation is illustrated in Figure 1. Notably, etiologies differed significantly between BC and NBC dogs (*p* = 0.009), although severity was not related to skull conformation (*p* = 0.26). Likewise, no significant association was found between etiology and severity (*p* = 0.147); however, descriptively, idiopathic, iatrogenic, and otitis‐related cases were more often linked to paralysis, whereas MUO‐related cases tended to present with paresis.

### 3.3 Ocular Abnormalities

**FIGURE 2:**
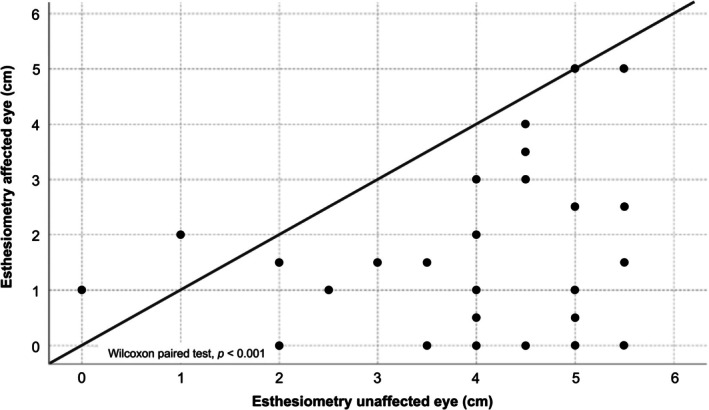
Scatterplot comparing corneal esthesiometry values between affected and unaffected eyes in dogs with facial nerve dysfunction. Each point represents an individual paired measurement. The diagonal line indicates equality between both eyes. Most values lie below this line, indicating reduced corneal sensitivity in affected eyes. This representation allows visualization of individual variability and paired differences between eyes. The difference was statistically significant (Wilcoxon paired test, *p* < 0.001).

**FIGURE 3:**
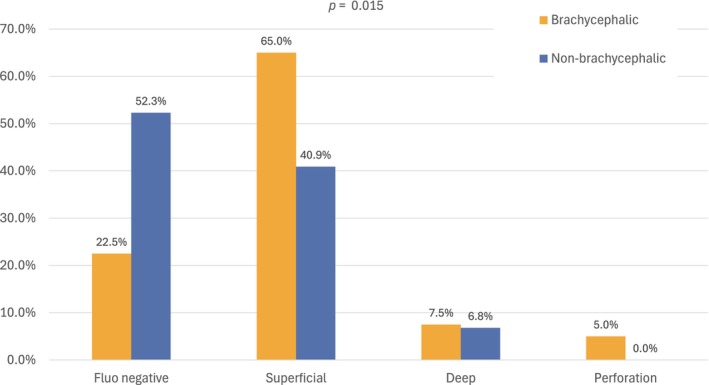
Distribution of corneal ulcer depth in brachycephalic (BC) and non‐brachycephalic (NBC) dogs. Superficial ulcers were the most common lesion in both groups but occurred more frequently in BC eyes (26 vs. 18). Deep stromal ulcers were equally represented (3 in each group), while corneal perforations were observed only in BC dogs (*n* = 2).

**Schirmer Tear Test**: The STT‐I was performed in 77 affected and 56 contralateral eyes. After excluding bilateral or preexisting ADDE/KCS, reduced STT‐I values were detected in 24 eyes (24/77; 31.2%) affected with FND, 11 of which (11/24; 45.8%) also exhibited xeromycteria. No significant difference was found between BC (9/37; 24.3%) and NBC (15/40; 37.5%) cases (*p* = 0.339). The etiologies included unknown (*n* = 13), MUO (*n* = 4), idiopathic (*n* = 2), otitis media/interna (*n* = 2), iatrogenic (*n* = 2), and intracranial neoplasia (*n* = 1), with no significant association with nKCS (*p* = 0.44). **Esthesiometry**: Corneal esthesiometry was measured in 40 affected eyes (16 BC and 24 NBC) and 31 contralateral eyes (11 BC and 20 NBC). Affected eyes showed significantly reduced sensitivity compared to contralateral eyes (*p* < 0.001) (Figure 2). Considering the affected eyes, no significant difference was observed between BC (0.50 cm [IQR: 0.00–2.50]) and NBC (1.25 cm [IQR: 1.00–2.25]) eyes (*p* = 0.391). Similarly, in contralateral eyes, corneal sensitivity did not vary significantly depending on skull conformation (BC [4.00 cm (IQR: 3.50–5.00)]; NBC [4.25 cm (IQR: 2.50–5.00)]) (*p* = 0.788). **Corneal findings**: Ulcerative keratitis was the most common ocular finding on admission, detected in 52 eyes (52/84; 61.9%), including 31 from BC (77.5%) and 21 from NBC (47.7%) dogs (*p* = 0.007). Among BC dogs, 83.9% of corneal ulcers were superficial, 9.7% were deep, and 6.4% were perforations. In NBC dogs, corneal ulcers were superficial in 85.7% of the cases and deep in 14.3%, with no perforations observed (Figure 3). **Intraocular findings**: Anterior uveitis (hypotony, miosis, flare and/or hypopyon) was identified in 25 eyes (25/84; 29.4%), with no significant difference between eyes from BC (14 eyes) and NBC (11 eyes) dogs (*p* = 0.348). Corneal ulceration was statistically associated with a higher incidence of uveitis (22/52; 42.3%; *p* < 0.001).

### 3.4 Treatment

Systemic therapy varied according to the underlying etiology, with antibiotics, prednisolone, and maropitant being the most prescribed. Topical therapy consisted mainly of lubricants (77/84; 91.6%), followed by broad spectrum antibiotics (55/84; 65.5%), cycloplegics/mydriatics (32/84; 38.1%), NSAIDs (28/84; 33.3%), and immunomodulators (14/84; 16.6%), with occasional use of heterologous serum, carbonic anhydrase inhibitors, or corticosteroids. Oral pilocarpine was administered in 12/74 patients (16.2%), in half of the cases combined with topical immunomodulators (6/74; 8.1%). Furthermore, bandage contact lenses were applied in 8/84 eyes (9.5%), five of which were from BC dogs.

Surgical treatments were recorded both at the initial visit and during follow‐up (Table 2). Surgery at presentation was performed in 19/84 eyes (22.6%). The most frequent procedure was lateral tarsorrhaphy (13 eyes; 15.5%), performed alone in 9 eyes (10.7%; 6 BC, 3 NBC), combined with corneal debridement in 3 eyes (3.6%; 1 BC, 2 NBC), or with amniotic membrane graft in 1 NBC eye (1.2%). Less frequent procedures included corneal debridement alone (2 BC eyes; 2.4%), enucleation (2 BC eyes; 2.4%), sutureless amniotic membrane graft combined with a nictitans flap (1 BC eye; 1.2%), and transpterygoid abscess drainage (1 BC eye; 1.2%).

**TABLE 2:** Summary of the most frequent surgical procedures performed at initial presentation or follow‐up periods in brachycephalic (BC) and non‐brachycephalic (NBC) patients.

Surgical procedure	Eyes (*n*)	Percentage (%)	BC dogs (*n*)	NBC dogs (*n*)
Lateral tarsorrhaphy	15	17.9	8	7
Enucleation	14	16.7	9	5
Corneal debridement	6	7.4	3	3
Corneal surgery (keratoplasty and AMG)	5	6.0	2	3

Abbreviation: AMG, amniotic membrane graft.

Additional surgical interventions were performed during follow‐up in cases with unsatisfactory clinical course. Enucleation was the most frequent procedure, performed in 12 eyes (14.3%; 7 BC, 5 NBC), usually due to progression of ulcerative keratitis to corneal perforation. Other interventions included an amniotic membrane graft followed shortly by a keratoplasty (1 BC eye; 1.2%), keratoplasty and nictitating membrane flap (2 NBC eyes; 2.4%), tarsorrhaphy (1 BC and 1 NBC eye; 2.4%), and cotton‐tip debridement (1 NBC eye; 1.2%).

Surgical intervention was significantly more frequent in BC dogs (24/40 [60.0%] vs. 15/44 [34.1%]; OR = 2.90, *p* = 0.021). Enucleation was performed in a total of 14 eyes (16.7%) and occurred more often in BC dogs (22.5% vs. 11.4%), although this difference was not statistically significant (*p* = 0.243).

### 3.5 Concurrent Diseases

A total of 46/74 dogs (62.2%) had concurrent diseases in addition to FND, including the primary disorders suspected to underlie the paralysis as well as unrelated systemic or neurologic conditions. The most frequent diagnoses were otologic and vestibular disorders (41), mainly vestibular syndrome (23) and otitis (17, including otitis externa, media, and interna), with one ear polyp also recorded. Inflammatory and immune‐mediated neurologic diseases accounted for 11 cases, including MUO (6). Other neurologic conditions comprised cranial polyneuropathy (4), epilepsy (2), and stroke (2). Infectious or inflammatory disorders were identified in 7 dogs, most commonly leishmaniasis (4), while neoplastic diseases were present in 6. Less frequent diagnoses included endocrine or metabolic disorders (4; hypothyroidism 3, exocrine pancreatic insufficiency 1), congenital malformations (3), immune‐mediated systemic diseases (3), atopic dermatitis (3), urogenital disorders (3), and cardiovascular disease (1).

Thyroxine (T4) and thyroid‐stimulating hormone (TSH) concentrations were measured in 12 dogs, including 3 with bilateral involvement, while T4 alone was assessed in 2 additional dogs (1 bilaterally affected). Decreased T4 concentrations accompanied by increased TSH levels were identified in 3 dogs (2 with bilateral involvement). In contrast, in the unilaterally affected dog, FND was ultimately attributed to otitis with associated meningitis and neuritis identified on MRI.

Ipsilateral vestibular syndrome was identified in 23/84 eyes (27.4%), affecting 14 BC (60.9%) and 9 NBC dogs (39.1%). In BC dogs, vestibular syndrome was attributed to idiopathic disease (6), otitis media/interna (5), and MUO (1), whereas in NBC dogs etiologies included MUO (3), and other less common causes (idiopathic, eosinophilic meningitis and stroke, 1 each). Etiology remained undetermined in 2 BC and 3 NBC dogs. A significant association was found between skull conformation and vestibular syndrome etiology (*p* = 0.011).

### 3.6 Follow‐Up Time and Outcome

Although follow‐up data were available for 68 eyes, information on facial function recovery was only recorded for 63. Among these, 30 eyes (47.6%) showed either complete (15/63; 23.8%) or partial recovery (15/63; 23.8%), including 11 BC and 19 NBC eyes. The median time to recovery was 155.95 days (95% CI: 63.3; 246.7) for BC dogs and 180 days (95% CI: 12.8; 347.2) for NBC dogs, without statistically significant difference (*p* = 0.542).

Motor function recovery was not significantly influenced by age (*p* = 0.687), skull conformation (*p* = 0.131), etiology (*p* = 0.336) or neuter status (*p* = 0.821). In contrast, both sex and laterality were significantly associated with outcome. Males were overrepresented in the non‐recovery group (72.7%), whereas females were more frequent among dogs achieving partial or complete recovery (53.3%; *p* = 0.043). Right‐sided involvement was also significantly associated with non‐recovery, accounting for 60.6% of affected dogs (*p* = 0.023). A tendency toward poorer outcomes was observed in dogs presenting with complete paralysis rather than paresis (72.7% vs. 50.0%; *p* = 0.075) and in those with relevant concomitant diseases such as leishmaniasis, hypothyroidism, or a history of trauma (*p* = 0.205). Notably, 69.7% of the dogs that failed to recover motor function had a concurrent systemic disease.

**FIGURE 4:**
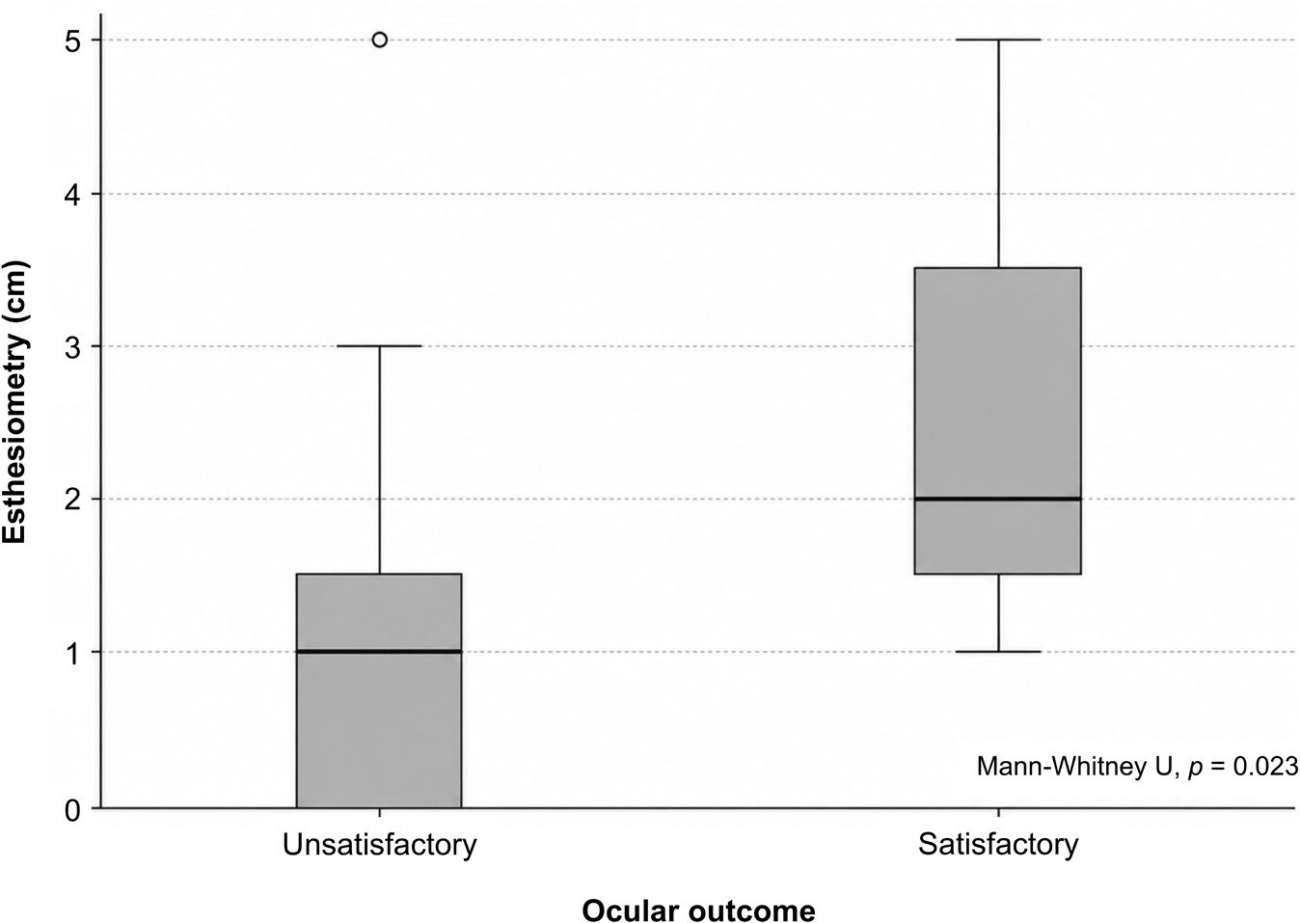
Comparison of corneal esthesiometry values between eyes with satisfactory and unsatisfactory ocular outcomes. Eyes that achieved a satisfactory outcome showed significantly higher corneal sensitivity (median 2 cm) compared to those with unsatisfactory recovery (median 1 cm; Mann–Whitney U test, *p* = 0.023).

Ocular recovery data were available for 55 eyes. Overall, satisfactory outcomes were achieved in 29.6% of BC eyes and 46.4% of NBC eyes, although this difference was not statistically significant (*p* = 0.269). Eyes that recovered demonstrated significantly higher corneal esthesiometry values compared with those that did not recover (2 cm vs. 1 cm; *p* = 0.023; Figure 4). The presence of uveitis was associated with a poorer prognosis, occurring in 44.1% of eyes without recovery versus 14.3% of eyes with partial or complete recovery (*p* = 0.037).

Among 18 eyes with nKCS, full tear production recovery was achieved in 7 eyes (38.9%). Of these, 4 eyes (22.2% of all nKCS eyes) recovered with pilocarpine alone, 1 eye (5.6%) with pilocarpine plus tacrolimus, 1 eye (5.6%) with cyclosporine A 0.2%, and 1 eye (5.6%) recovered without specific therapy. Enucleation was ultimately required in 7 eyes (38.9%).

Regarding the 52 eyes with ulcerative keratitis, 22 (42.3%) healed, 12 (23.1%) worsened, and the outcome was unknown in 16 (30.8%). Two eyes were enucleated immediately after the diagnosis of corneal perforation, and an additional 8 required enucleation during the follow‐up period. In total, 10 eyes (19.2%) with ulcerative keratitis underwent enucleation.

Overall, 26 patients (35.2%) experienced the most severe consequences of the disease, and 14 eyes (17.6%) ultimately required enucleation (9 in BC dogs [22.5%] and five in NBC dogs [11.4%]; *p* = 0.243). In addition, 10 patients (13.5%) were euthanized, and 2 (2.7%) died during follow‐up for reasons unrelated to the study.

## 4 Discussion

To the authors' knowledge, this study provides the first long‐term characterization of ocular findings in dogs affected by FND. Ocular surface disease was highly prevalent, particularly in BC dogs, and a substantial proportion of cases progressed to severe, often irreversible complications.

As in human medicine, idiopathic disease is the most common cause of FND in dogs [3, 6, 39, 40]. In people, Bell's palsy accounts for most cases of acute, nontraumatic facial paralysis and is characterized by sudden unilateral peripheral nerve dysfunction of unknown origin [39]. In a recent canine study, idiopathic paralysis represented 29.5% of all cases and 36.7% of naturally occurring disease [3]. In our cohort, the lower prevalence (14.9%) likely reflects stricter diagnostic criteria and exclusion of cases lacking a complete diagnostic work‐up.

A significant association was found between skull conformation and etiology. In BC dogs, otitis media/interna and idiopathic causes predominated, whereas NBC dogs were more commonly affected by central disorders. Previous studies have reported a strong association between otitis and FND in BC dogs. In French Bulldogs undergoing subtotal ear canal ablation and lateral bulla osteotomy, preoperative FND has been described in up to 75% of ears affected by chronic otitis [41]. TECA‐LBO carries a considerable risk of iatrogenic facial nerve injury due to surgical exposure of the nerve [42, 43]. In our study, iatrogenic injury and otitis media/interna accounted for 13.5% and 12.2% of cases, respectively, and were more common in BC dogs. Consistent with previous reports describing narrower ear canals and increased susceptibility to middle ear disease in these breeds [44], the French Bulldog was the most frequently affected breed in our cohort.

Although no statistically significant association was identified between FND severity and either skull conformation or etiology, certain trends were observed. Idiopathic, otitis‐related, and iatrogenic cases more commonly resulted in complete paralysis, whereas cases associated with MUO tended to present as paresis. These differences likely reflect distinct pathogenic mechanisms. In idiopathic cases, the lesion is thought to occur in the peripheral nerve or its nucleus, often leading to complete motor deficit [45]. As mentioned before, otitis and ear surgery may result in severe injury or even nerve transection in some cases [24, 42, 46]. By contrast, MUO is a CNS inflammatory disorder that rarely causes direct peripheral nerve damage [47, 48, 49], which could explain why paresis rather than paralysis is more common. These findings suggest that the anatomical location and nature of the lesion may play a greater role in determining clinical severity than skull conformation. However, these observations should be interpreted cautiously given the small size of the etiologic subgroups.

Approximately one third of eyes with FND showed STT‐1 values consistent with nKCS. Previous veterinary reports approached the question from the opposite direction, describing FND in 38% of dogs diagnosed with nKCS [4]. Neurogenic KCS results from damage to the parasympathetic fibers of the facial nerve proximal to the geniculate ganglion. In our cohort, nearly half of these cases showed ipsilateral xeromycteria, suggesting injury proximal to the pterygopalatine ganglion. Despite this relatively high prevalence, this finding was not significantly associated with skull conformation or etiology.

Given the role of the trigeminal nerve in reflex lacrimation, some authors have implicated corneal hypoesthesia secondary to trigeminal nerve injury in the pathogenesis of nKCS [50]. In our study, reduced corneal sensitivity was frequently observed in dogs with FND. This may reflect concurrent facial and trigeminal nerve dysfunction due to central or inflammatory processes, or secondary neurotrophic changes resulting from chronic corneal exposure. No association was found between corneal sensitivity loss and etiology, although esthesiometry results should be interpreted cautiously as impaired blinking may lead to overestimation of hypoesthesia. These findings support the routine use of STT‐1 and corneal esthesiometry in the evaluation and follow‐up of dogs with FND.

Ulcerative keratitis was the most common ocular abnormality at presentation and was significantly more frequent and severe in BC dogs, consistent with previous epidemiological data [51]. In the context of FND, this condition is best classified as neuroparalytic keratitis, and the increased susceptibility of BC dogs to deep ulcers and secondary uveitis likely contributes to their poorer ocular prognosis [52].

Treatment of FND depends on the underlying etiology, and discussion of therapeutic approaches is beyond the scope of this study. In human medicine, Bell's palsy is thought to result from facial nerve inflammation and edema, most often secondary to herpes simplex virus type 1 or herpes zoster virus [39], and is typically treated with corticosteroids, sometimes combined with antivirals, although the latter have not shown clear benefits in recovery rates [53]. Unfortunately, no effective treatment is currently available in canine patients with idiopathic FND [13]. Consequently, therapy focuses primarily on ocular surface protection and management of complications. Medical treatment is usually the first approach, but surgical intervention is often required.

Ocular lubrication is essential as long as eyelid motor function remains incomplete, and any corneal ulcer that develops must be promptly treated. Management of nKCS should include oral or topical pilocarpine [4, 5, 50]. In our study, oral pilocarpine was prescribed in 12 patients, combined with immunomodulatory drugs in 6 of them. Of those, 41.7% showed increased tear production, comparable to the 48% resolution rate reported in Galley's study [4].

Nearly one quarter of eyes required surgery at presentation, most commonly lateral tarsorrhaphy. This underscores the limited efficacy of medical management alone, particularly in BC dogs, which accounted for more than half of the initial surgical cases. Additional procedures were often required during follow‐up, and enucleation was ultimately performed in 16.7% of eyes. While not statistically significant, the higher proportion of enucleations in BC dogs emphasizes the impact of conformational factors on prognosis. The variety of surgical techniques used reflects the complexity of management, similar to reports in human and veterinary medicine where procedures such as tarsorrhaphy or eyelid loading are commonly performed to protect the cornea in FND [54, 55, 56]. More advanced reconstructive techniques described in human medicine, such as hypoglossal–facial anastomosis [57], have not yet been reported in veterinary patients.

While idiopathic disease is reported as the most frequent cause of peripheral vestibular syndrome [58], otitis media/interna is also an important contributor [58, 59]. The frequent concurrence of facial and vestibular dysfunction is explained by the close proximity of the facial and vestibulocochlear nerves [2, 60]. In our study, recovery tended to be poorer in patients with concomitant systemic or neurologic disease, consistent with previous reports suggesting that comorbidities may negatively affect prognosis [60].

Overall, fewer than half of the cases recovered from FND, confirming the often irreversible nature of this condition. Outcome was not significantly influenced by age or skull conformation, although recovery tended to be less frequent in BC dogs. Interestingly, female sex was associated with a higher likelihood of recovery, although this finding should be interpreted cautiously given the limited sample size. While formal statistical evaluation was not possible, prognosis also seemed to be shaped by etiology, with MUO‐related cases showing the most favorable outcomes and otitis‐ associated cases rarely improving. Less than half of the idiopathic cases recovered, similar to previous veterinary studies [3, 60], but in contrast to the favorable prognosis for human Bell's palsy [39]. Though not statistically significant, recovery was more likely in dogs with paresis than with paralysis, suggesting that partial nerve integrity favors regeneration, as previously reported [39].

Right‐sided paralysis was associated with poorer recovery than left‐sided involvement, an unexpected finding that may reflect the limited sample size. However, right‐sided cases were more frequently associated with otitis and iatrogenic injury. Although differences in outcome according to laterality have not been previously reported, surgical literature suggests that anatomical and procedural factors related to side may influence surgical complexity and potentially affect outcomes [61].

Ocular outcome was significantly influenced by corneal sensitivity, with higher sensitivity observed in eyes that recovered. This is consistent with the recognized role of corneal innervation in maintaining epithelial metabolism and integrity. Reduced corneal sensitivity has been associated with impaired healing and neurotrophic keratopathy in both human and veterinary medicine [37, 62].

Uveitis was associated with poorer ocular outcomes. BC dogs, which showed a higher proportion of uveitis, are known to be predisposed to ocular surface disease, as summarized exquisitely by Sebbag and colleagues [63]. Although the difference in outcome between BC and NBC patients was not significant, their increased tendency toward both ulcerative disease and intraocular inflammation may explain the trend toward less favorable ocular recovery in BC breeds.

More than one third of patients developed irreversible complications, and nearly one fifth required enucleation. Although not statistically significant, enucleation was almost twice as frequent in BC dogs, emphasizing the need for early medical treatment, timely surgical intervention, and close monitoring in this population.

This study has several limitations inherent to its retrospective design. Incomplete clinical records and small sample sizes within etiologic subgroups limited the availability of outcome data and the statistical power of subgroup analyses. The absence of advanced imaging in many patients restricted the ability to fully determine the underlying cause of dysfunction. Assessment of the corneal reflex was also challenging, as impaired blinking may have led to underestimation of esthesiometry values, despite careful attention to globe retraction and head withdrawal during corneal stimulation. In addition, the heterogeneity of treatments prevented standardized evaluation of efficacy. Finally, as this was a referral hospital population, case selection bias cannot be excluded, and the findings may not fully represent the broader canine population.

In conclusion, FND in dogs carries a guarded prognosis, with fewer than half of the cases regaining motor function and over one third experiencing irreversible consequences. Ocular morbidity, particularly nKCS and neuroparalytic ulcerative keratitis, often led to poor outcomes despite therapy, especially in BC dogs. These findings highlight the importance of early diagnosis, aggressive medical management, timely surgical intervention in high‐risk cases, and close follow‐up to prevent vision‐threatening complications.

## Author Contributions

**J. Ríos:** methodology, writing – review and editing. **L. Gaztelu:** resources, data curation, formal analysis, writing – review and editing, methodology, conceptualization, investigation, writing – original draft. **M. Leiva:** conceptualization, writing – review and editing, supervision. **T. Peña:** supervision, writing – review and editing.

## Conflicts of Interest

The authors declare no conflicts of interest.

## Data Availability

The data that support the findings of this study are available from the corresponding author upon reasonable request.
